# Genetic markers in oligodendroglial tumours

**DOI:** 10.2478/v10019-010-0007-y

**Published:** 2010-03-18

**Authors:** Tomaz Velnar, Uros Smrdel, Mara Popovic, Gorazd Bunc

**Affiliations:** 1 Department of Neurosurgery, University Medical Centre Maribor, Maribor, Slovenia; 2 Institute of Oncology Ljubljana, Ljubljana, Slovenia; 3 Department of Pathology, Medical Faculty Ljubljana, Ljubljana, Slovenia

**Keywords:** oligodendroglioma, genetic markers, chemotherapy, prognosis

## Abstract

**Background:**

Oliogodendrogliomas are brain tumours composed of the cells resembling oligodendrocytes. They represent the third most common glial tumour, comprising 2.5% of all primary brain tumours and 5–20% of all gliomas.

**Conclusions:**

Oligodendroglial tumours with 1p and 19q loss demonstrate a better overall prognosis due to more indolent clinical behaviour and higher sensitivity to treatment. Additionally, 1p and 19q loss is a marker of clinical utility, helping to assess tumour sensitivity to chemotherapy and harbouring the potential for improving the diagnosis and survival of oligodendroglioma patients as well as future clinical practice.

## Introduction

Over the past decade, a remarkable progress in the aspects of cancer treatment was made.^1^ Advances in imaging methods, molecular biology, surgical and chemotherapeutic techniques and radiation delivery have all improved the prognosis of cancer patients more than ever before.^1^–^6^ Despite optimal diagnostics and treatment, cancer burden in the society is still ranking too high.

After cardiovascular disease, cancer is the second leading cause of morbidity and mortality worldwide.^7^ The most frequent brain tumours are gliomas. According to cell morfology they may be divided into astrocytomas, oligodendrogliomas, ependymomas and oligoastrocytomas.^4^,^8^ Due to a favourable response to chemotherapy, oligodendrogliomas have gained much interest in the past decade. They may be additionally subdivided into prognostic subgroups according to the histopathology, molecular and biological characteristics. The genetic alterations in tumour cells, together with clinical and histolpathological properties, may all define the most appropriate therapy and predict the outcome of the treatment.^4^,^9^

## Epidemiology and aetiology

Oliogodendroglial tumours arise from oligodendroglial cells or immature glial precursors and represent the third most frequently encountered type of glial tumours, ranking after glioblastoma and astrocytomas. They constitute 5–20% of all gliomas and 2.5% of all primary brain tumours.^4^,^9^–^11^ About 1500 new cases are diagnosed in Europe, and in Slovenia there are about 20 cases each year.^12^,^13^ The annual incidence rate of oligodendrogliomas is two to four per 1,000,000 people and the incidence is increasing every year.^4^,^9^ It has significantly increased over the past years, but this may primarily be due to the use of additional diagnostic criteria in recent years.^4^,^9^,^11^,^12^

Although oligodendrogliomas may occur at any age, there is a peak incidence between 40 and 45 years.^4^,^11^ In spite of, the brain tumours in children are very frequent^14^, oligodendrogliomas are rare and representing 2% of all brain tumours in patient younger than 14 years.^11^ Male-to-female ratio is 1.5 to 1. In males, peak incidence was described between 45 to 49 years and in females between 55 to 59 years.^4^,^9^ In younger patients, low grade oligodendrogliomas predominate. Familial clustering of tumours was found, with neither genetic factors nor special pattern of inheritance.^15^–^17^ Moreover, causes for oligodendroglioma evolvement are unknown, no lifestyle or environmental factors are discovered, even though rare individual cases of oligodendroglioma in patients previously irradiated for other reasons have been documented.^9^,^11^,^15^,^18^ More than 90% of oligodendrogliomas arise supratentorially in the cerebral white matter, predominantly in the frontal lobes, but patients have been reported with oligodendroglima in basal ganglia, posterior fossa, or spinal cord. Supratentorial locations according to tumour frequency are as follows: 55% in frontal lobes, 47% in temporal, 20% in parietal and 4% in occipital lobes.^4^,^9^,^17^

## Pathology and neurooncology

Oligodendrogliomas exhibit an infiltrative growth pattern, although not to such an extent as astrocytomas.^4^,^19^,^20^ According to the growth pattern and histological characteristics, two grades of malignancy are distinguished by the WHO: well differentiated oligodendroglioma of grade II and anaplastic oligodendroglioma of grade III.^17^,^19^,^21^ The latter may evolve from a low-grade oligodendroglioma, becoming gradually more anaplastic over time, or present *de novo*, without a preceding low-grade tumour. There are 77% of low-grade and 23% of anaplastic oligodendrogliomas.^4^,^9^,^21^ According to the tumour cell morphology, two types have been described: pure oligodendrogliomas and mixed gliomas or oligoastrocytomas, containing neoplastic cells of oligodendroglial and astroglial phenotype.^4^,^13^,^21^–^23^ Oligodendrogliomas may sometimes invade meninges.^4^ Very rarely, the tumours may metastasize to other locations, such as lung, liver, bone and cervical lymph nodes.^24^,^25^ Although extremely rare, metastatic disease is encountered more frequently due to the improved survival of oligodendroglioma patients.^4^,^9^

Because no specific immunohistological markers for oligodendrogliomas exist, the histological diagnosis may be challenging for a pathologist.^9^ However, a chromosomal alteration has been reported, which is the most common lesion found in oligodendroglial tumours and involves a deletion at chromosomal loci 1p and 19q. A combined loss of 1p and 19q identifies a group of good prognosis tumours and has been reported, depending on the literature, in the range of 50% to 90% or 60% to 70% of oligodendrogliomas of any grade. On the other hand, the incidence of either 1p or 19q deletions alone is 75%.^4^,^9^,^17^,^26^,^27^

## Tumour signs and symptoms

The symptoms of oligodendroglial tumours are similar to other primary and solitary secondary brain neoplasms^28^,^29^, with epileptic seizures being the most common symptom, presenting in 35% to 85% of patients.^4^ Seizures may be generalised, simple or complex partial, or a combination of these. They may be experienced for a number of years before the diagnosis.^30^ Other symptoms include headaches, sensory and motor disturbances in terms of localised limb weakness, sudden or insidious change in personality and mood, visual complaints, nausea and dizziness. Symptoms usually precede the definitive diagnosis for 2.9 months to 5 years.^4^,^31^,^32^

## Oligodendroglioma treatment

There are three therapeutic modalities for the treatment of oligodendrogliomas that are connected and combinable: surgery, radiation therapy and chemotherapy.^4^,^9^ All three are often used successively. Surgery remains a most frequently employed method both in order to perform a tumour reduction or a gross resection where possible and to obtain tissue samples for the definite diagnosis.^9^,^33^ The resection decreases the tumour mass effect on the brain with concomitant neurological consequences and reduces the tumour load during radiotherapy, which is the next and often the following form of the treatment in grade 3 tumours.^24^,^35^ Radiotherapy is used due to an invasive nature of tumour growth where a deep infiltration of tumour cells cannot be determined during surgery and, therefore, prevents a complete removal. As a consequence, the disease relapses slowly but inevitably.^35^,^36^ A tumour relapse after a removal, which was not possible to be detected by clinical means, is termed a recurrence. It takes place at the operative site in the form of a high grade tumour, an anaplastic oligodendroglioma or even glioblastoma. While low-grade tumours may recur after many years, anaplastic ones tend to do so sooner.^4^

The third option of the treatment is chemotherapy, which is being widely used, again for grade 3 tumours. The most frequently employed agents are procarbazine, vincristine, and lomustine (CCNU) (PCV scheme).^37^–^39^ It has been reported that 60% to 75% of patients respond to PCV chemotherapy with 10–32 months of median response duration.^9^ Chemotherapy is used as a treatment option and with or without radiotherapy, the latter option in children, where radiation is usually withheld due to adverse effects on the developing nervous system. Chemotherapy application before radiotherapy is becoming a standard practice also in adults in order to spare the side effects of radiation and to have a second line of the treatment option in case of tumour progression with comparable time to progression and overall survival.^4^,^37^–^40^

Besides standard PCV chemotherapy, temozolomide is being widely used as an alternative or supplement treatment both in primary and in metastatic disease.^10^,^41^–^43^ In comparison to PCV chemotherapy, there are fewer side effects reported and the therapy regimen is more convenient.^38^,^40^,^44^–^46^ On the contrary to low-grade oligodendrogliomas, where radiation is delayed until tumour progression, patents with anaplastic oligodendrogliomas receive both radiation and chemotherapy and this combination is superior to either treatment alone.^4^,^9^,^34^,^35^ Other chemotherapeutic agents used are carboplatin, cisplatin, etoposide, melphalan, thiotepa and other nitrosourea drugs, as well as interferon-β and recently bevacizumab.^47^ A reason for an increase of chemotherapy comes from the observations that low-grade and anaplastic oligodendrogliomas are chemosensitive tumours.

Many genetic abnormalities are encountered in brain neoplasm and many of these identified may emphasize potential diagnostic, therapeutic and prognostic implications.^9^,^40^

## Genetic markers in oligodendrogliomas

Various genetic markers have been described in connection to oligdendroglial tumours and are briefly discussed below.

### Chromosomes 1 and 19

As already stated, abnormalities in chromosomes 1 and 19 are the most significant. 1p and 19q losses are encountered in 80% to 90% of grade 2 and in 50% to 70% of grade 3 oligodendrogliomas.^17^ On the contrary, childhood oligodendrogliomas only rarely exhibit chromosomal abnormalities. In adults, in the majority of cases chromosome losses involve the entire long arm of chromosome 19 and are present in connection with losses from chromosome 1p. They were observed to be more common in frontal, occipital and parietal lobes than in temporal lobe tumours.^48^,^49^

### Methylation of MGMT genes

Another common finding in oligodendrogliomas is methylation of DNA regions that code for MGMT genes, present in approximately 93% of cases. The end result is transcriptional silencing of genes responsible for DNA repair enzyme, which may contribute to higher chemosensitivity.^17^,^49^

### Mutations in p53 gene

Mutations in p53 gene are described in 10% to 15% of tumours without 1p and 19q loss.^17^ Such tumours arise most commonly in the temporal lobes; histologically they are anaplastic or mixed oligoastrocytomas and express poor chemosensitivity.^50^,^51^ Response rate or efficacy of the chemotherapy treatment was observed only in 33% of patients with p53 mutation and intact 1p and 19q chromosomes, as opposed to tumours with intact p53 gene and 1p and 19q or only 1p mutation, where the response rate was 100%.^17^,^50^

### Growth factors and other genetic abnormalities

Growth factors overexpression includes epidermal growth factor receptor (EGFR), vascular endothelial growth factor (VEGF) and platelet-derived growth factor (PDGF).^17^,^52^ EGFR overexpression was observed in 50% of oligodendrogliomas; the percentage of other two overexpressed factors is somewhat lower. Other chromosomal abnormalities consist of genetical abnormalities or losses from chromosomes 10q and 9p. They are preferentially encountered in anaplastic oligiodendrogliomas without of 1p and 19q loss.^17^,^48^ Additionally, oligodendrocyte transcription factors, such as Olig 1 and Olig 2 that may be used as markers of oligodendrogliomas, are highly expressed in oligodendrogliomas, as well as in astrocytomas.^17^,^53^,^54^

## Prognostic and diagnostic value of 1p and 19q abnormalities

Pure oligodendrogliomas show a better prognosis than astrocytomas of the same grade and oligoastrocytomas are prognostically in between the former two.^9^ For the management of tumours and for prognostic and therapeutic decisions, it is important to identify the tumour type correctly.^23^ Microscopical appearance, which forms the basis for the distinction of gliomas, is not always as clear as to set the diagnosis directly. It is sometimes particularly difficult to distinguish oligodendrogliomas and oligoastrocytomas. In literature studies, the diagnostic concordance observed in these tumours may range from 52% to 86% among pathologists.^48^ This fact necessitated a search for an additional diagnostic tool for the oligodendrogliomas. Two factors influence the difficulties in histopatological diagnostics: (1) lack of a specific immunohistochemical cell marker for oligodendroglial tumours and (2) a variation in tumour microscopic morphology. Genetically, a combined loss of 1p and 19q is typical for oligodendrogliomas and rare in gliomas of other type, while isolated 19q loss occurs in mixed oligoastrocytomas and in astrocytomas.^48^,^49^,^55^ Chromosome 1p and 19q status may be assessed by a variety of techniques, such as microsatellite analysis, fluorescence in situ hybridization (FISH), genomic hybridization and quantitative polymerase chain reactions.^17^,^48^,^55^ Besides being a valuable diagnostic marker due to its specificity, it was discovered that 1p and 19q loss also acts as a powerful marker in the prognosis of the disease and as a predictor of chemotherapeutic response and survival.^42^,^48^,^56^ The follow-up period described in the studies varies from two to five years.^23^,^57^ This is somehow short, when one takes into account the survival period in oligodendroglioma, which varies between four to seven years, depending on the grade. Oliogodendrogliomas harbouring 1p and 19q deletion behave more indolently and respond favourably to PCV chemotherapy and temozolomide as well as to radiotherapy.^42^,^55^ For example, the reported correlation between 1p and 19q loss and PCV regimen in the treatment response ranged from 93% to 100%. Temozolomide as a replacement for PCV therapy, due to a better toxicity profile, showed 46% to 55% response rate to the treatment. Also, time to progression of the disease correlated with 1p and 19q loss.^9^,^58^,^59^ On the other hand, the therapeutic sensitivity of 1p and 19q-intact tumours is less favourable and the survival is therefore shorter.^58^,^60^,^61^

Another factor reported to bear the prognostic significance is o6-methylguanine-DNAmethyltransferase (MGMT), an enzyme involved in DNA repair.^62^,^63^ In many tumours, including gliomas, alterations in DNA may be found, such as methylation of the promoter region and their genes. Methylated DNA is less readily accessible to transcription factors and results in the loss of gene function. As MGMT is one of the key factors in resistance to chemotherapy, hypermethylation inhibits the repair mechanism due to a lower level of the active enzyme.^6^,^17^,^58^,^63^ MGMT methylation rates in oligodendrogliomas range from 25% to 85% and were reported to be strongly associated with 1p and 19q loss.^58^,^59^ However, the response rate to chemotherapy and time to progression of oligodendrogliomas were not observed to be in correlation with the degree of MGMT methylation, as is the case with glioblastoma, where promoter methylation correlated with response to the alkylating agent treatment and survival. The cause probably lies in different genes and pattern of promoter methylation, which is present in astrocytic cells.^17^,^58^,^62^–^64^

1p and 19q deletion is a predictive factor of tumour response principally to chemotherapy, and radiotherapy as well.^48^,^59^–^61^ A number of centres employ evaluation of 1p and 19q status as a laboratory test, which is used in conjunction to clinical status, imaging and patohistological diagnosis for predicting the patient response to the treatment. This enables to tailor the most effective and appropriate therapy for the individual patient.^23^,^55^,^58^ However, there are still unexplained issues in connection to 1p and 19q loss. To begin with, the genes and their exact functions in the pathogenesis of oligodendrogliomas, located on the long arms of chromosomes 1 and 19, need to be identified for some patients with 1p and 19q intact tumours which respond well to the therapy and vice versa.^9^,^58^ Despite the fact that 1p and 19q status helps in selecting patients with respect to therapeutic regimen, there were no revolutionary improvements in the treatment outcomes.^55^ A further investigation is required in order to elucidate the unsolved questions in oligodendroglioma biology.

## Conclusions

Oligodendroglial tumours with 1p and 19q loss demonstrate a better overall prognosis due to a more indolent clinical behaviour and higher sensitivity to treatment. The 1p and 19q status acts as a prognostic marker, since its loss is associated with an improved outcome compared to non-1p and 19q deleted oligodendrogliomas and astrocytomas of a same grade. 1p and 19q testing proved to be particularly useful for determining the tumour type in morphologically ambiguous cases, as it acts as a valid marker of classical oligodendroglial tumours, when present. Additionally, 1p and 19q loss is a marker of clinical utility, helping to assess tumour sensitivity to chemotherapy and harbouring the potential for improving the diagnosis and survival of oligodendroglioma patients as well as future clinical practice.
